# Alloantigen-Induced Regulatory T Cells Generated in Presence of Vitamin C Display Enhanced Stability of Foxp3 Expression and Promote Skin Allograft Acceptance

**DOI:** 10.3389/fimmu.2017.00748

**Published:** 2017-06-28

**Authors:** Eirini Nikolouli, Matthias Hardtke-Wolenski, Martin Hapke, Michael Beckstette, Robert Geffers, Stefan Floess, Elmar Jaeckel, Jochen Huehn

**Affiliations:** ^1^Department Experimental Immunology, Helmholtz Centre for Infection Research, Braunschweig, Germany; ^2^Department Gastroenterology, Hepatology, Endocrinology, Hannover Medical School, Hannover, Germany; ^3^Genome Analytics, Helmholtz Centre for Infection Research, Braunschweig, Germany

**Keywords:** allo-iTregs, Foxp3, vitamin C, demethylation, transplantation

## Abstract

Regulatory T cells (Tregs) are critical for the maintenance of immune homeostasis and self-tolerance and can be therapeutically used for prevention of unwanted immune responses such as allotransplant rejection. Tregs are characterized by expression of the transcription factor Foxp3, and recent work suggests that epigenetic imprinting of *Foxp3* and other Treg-specific epigenetic signatures genes is crucial for the stabilization of both *Foxp3* expression and immunosuppressive properties within Tregs. Lately, vitamin C was reported to enhance the activity of enzymes of the ten-eleven translocation family, thereby fostering the demethylation of *Foxp3* and other Treg-specific epigenetic signatures genes in developing Tregs. Here, we *in vitro* generated alloantigen-induced Foxp3^+^ Tregs (allo-iTregs) in presence of vitamin C. Although vitamin C hardly influenced the transcriptome of allo-iTregs as revealed by RNA-seq, those vitamin C-treated allo-iTregs showed a more pronounced demethylation of *Foxp3* and other Treg-specific epigenetic signatures genes accompanied with an enhanced stability of *Foxp3* expression. Accordingly, when being tested *in vivo* in an allogeneic skin transplantation model, vitamin C-treated allo-iTregs showed a superior suppressive capacity. Together, our results pave the way for the establishment of novel protocols for the *in vitro* generation of alloantigen-induced Foxp3^+^ Tregs for therapeutic use in transplantation medicine.

## Introduction

Regulatory T cells (Tregs) play an important role for the maintenance of immune homeostasis and self-tolerance (1, 2). At the same time, Tregs are very promising immunotherapeutic candidates to prevent unwanted immune responses like autoimmune disorders or allotransplant rejections, and first clinical trials show encouraging data regarding the use of Tregs as a cellular therapy (3–11).

Tregs are characterized by the expression of the transcription factor forkhead box P3 (Foxp3) (12, 13). Foxp3 is not only a marker for Tregs, but also critically required for their suppressive capacity (12–15). Loss of Foxp3 expression or function can lead to severe autoimmune disorders (16, 17), making the stability of Foxp3 expression a critical issue, which has to be ensured before employing Foxp3^+^ Tregs in therapeutic applications (18).

*In vitro* generation of polyclonal or alloantigen-specific Foxp3^+^ Tregs can be easily achieved by appropriate TCR stimulation of conventional Foxp3^−^CD4^+^ T cells in presence of TGF-β and IL-2 (*in vitro* induced Tregs, iTregs) (19). However, these iTregs display an unstable Treg phenotype and rapidly lose Foxp3 expression and suppressive activity upon *in vitro* re-stimulation in the absence of TGF-β or upon adoptive transfer (20–22). Thus, clinical use of iTregs remains critical as these cells might even convert into effector T cells, potentially exerting negative effects for the patient.

Previous work from others and us demonstrated that the stability of Foxp3 expression is under epigenetic control. In particular, a CpG-rich evolutionary conserved element within the first intron of the *Foxp3* locus, which is called Treg-specific demethylated region or conserved non-coding sequence 2, is selectively demethylated in *ex vivo* isolated Foxp3^+^ Tregs, but methylated in both *ex vivo* isolated conventional Foxp3^−^ T cells as well as iTregs (20, 22–24). Demethylation of the TSDR is not mandatory for the initiation of Foxp3 expression, but rather linked to its long-term maintenance (15, 20, 22, 25, 26). Although stable Foxp3 expression is essential for ensuring the suppressive activity of Tregs, it is not sufficient to confer and maintain the full Treg phenotype. Instead, a number of additional Treg-specific epigenetic signature genes, including *Ctla4, Eos, Gitr*, and *Helios*, have to be demethylated in order to ensure lineage stability and fully functional Foxp3^+^ Tregs (22, 27, 28).

Demethylation of the TSDR and the other Treg-specific epigenetic signature genes is initiated during early stages of thymic Treg development (29, 30). We could recently demonstrate that thymic antigen-presenting cells (APCs), including thymic dendritic cells and medullary thymic epithelial cells, have unique functional properties that foster the acquisition of a Treg-specific epigenetic signature in developing Tregs (31). This process is achieved through an active mechanism involving enzymes of the ten-eleven translocation family (TETs) (29, 30, 32–34). TET enzymes catalyze the oxidation of 5-methylcytosine (5mC) to 5-hydroxymethylcytosine (5hmC), which is the initiating step of active DNA demethylation (35–37). A cofactor, which was recently found to foster this process, is the nutrient vitamin C, as it can enhance the enzymatic activity of TET proteins (30, 38–40). More specifically, vitamin C-induced changes in the DNA 5hmC levels are suppressed in *Tet1/2* double knockout embryonic stem cells and vitamin C acts synergistically with retinoic acid (RA) to modulate TET enzymes through enhancing the recirculation of Fe^2+^ and activation of TET2 and TET3 transcription, respectively (38, 41). Recently, a direct connection of vitamin C with the generation of stable Tregs was reported, as it promotes TSDR demethylation in a Tet2/Tet3-dependent manner, thereby increasing stability of Foxp3 expression in polyclonal iTregs (30, 42). Accordingly, the originally demethylated TSDR within peripheral Tregs showed an increase in methylation after treatment with the sodium-dependent vitamin C transporter inhibitor, sulfinpyrazone (42).

On the basis of these observations, we here exploited the special properties of vitamin C to support the *in vitro* generation of stable, alloantigen-induced Tregs (allo-iTregs) with long-term suppressive activity under clinically relevant conditions. While our data revealed that addition of vitamin C to the alloantigen-specific Treg induction cultures did not result in overt changes in the transcriptomes of allo-iTregs, we could observe the acquisition of Treg-specific methylation patterns along with enhanced stability of Foxp3 expression in vitamin C-treated allo-iTregs. Importantly, these stabilized allo-iTregs showed superior suppressive capacity when tested *in vivo* in a highly immunogenic skin transplantation model, suggesting that vitamin C can support the *in vitro* generation of Tregs with long-term suppressive activity for therapeutic use in transplantation medicine.

## Materials and Methods

### Mice

BALB/c, Foxp3^RFP^ reporter mice (C57BL/6 background) (43), and congenic CD45.1 Foxp3^hCD2^ reporter mice (C57BL/6 background) (15) were bred and maintained under specific pathogen-free conditions in the animal facility of the Helmholtz Centre for Infection Research (Braunschweig, Germany). Rag2^−/−^ mice were bred and maintained under specific pathogen-free conditions in the animal facility of Hannover Medical School (Hannover, Germany). All mice were used at the age of 6–10 weeks. The animal experiments were approved by the Niedersächsisches Landesamt für Verbraucherschutz und Lebensmittelsicherheit (LAVES): animal licensing committee permission no. 10/0071 and 15/1878. All experiments were performed in accordance with regulations according to FELASA, and animals were handled with care and welfare.

### Antibodies and Flow Cytometry

Cell suspension from lymph nodes and spleen were collected and labeled directly with fluorochrome-conjugated anti-mouse CD3ε (145-2C11), CD4 (RM4-5), CD8α (53-6.7), CD11c (N418), CD19 (6D5), CD25 (PC61.5), CD49b (DX5), CD90.2 (53-2.1), CD45.1 (A20), F4/80 (BM8), and anti-human CD2 (RPA-2.10). For exclusion of dead cells, the Fixable Blue Stain Kit (Invitrogen) was used. Flow cytometric analysis was performed on LSRII or LSR-Fortessa (BD Biosciences), and data were analyzed with FlowJo software (Tree Star).

### Isolation of Peripheral T Cells

For the *in vitro* assays, peripheral CD4^+^ T cells were enriched from pooled spleen and lymph node cells from Foxp3^RFP^ reporter mice (C57BL/6) using direct anti-CD4 (L3T4) microbeads followed by magnetic separation using the autoMACS separation system (Miltenyi Biotec). Subsequently, enriched cells were labeled with respective antibodies and sorted as CD4^+^CD90.2^+^Foxp3^RFP−^ peripheral T cells on ARIA II (BD Biosciences) or MoFlo (Beckman Coulter). For the *in vivo* assay, naïve CD4^+^ T cells were isolated from pooled spleen and lymph node cells from Foxp3^hCD2^ CD45.1 reporter mice. Briefly, CD4^+^ T cells were enriched as described above and labeled with respective antibodies. Subsequently, naïve CD4^+^ T cells (CD4^+^CD90.2^+^CD45.1^+^CD25^−^CD44^−^CD62L^hi^Foxp3^hCD2−^) were sorted on ARIA II or MoFlo.

### Isolation of Splenic Dendritic Cells (sp-DCs)

To isolate sp-DCs, spleens from BALB/c mice were collected, finely chopped into pieces and digested in complete RPMI 1640 medium (Life Technologies), containing 0.2 mg/ml collagenase/dispase (Roche) and 0.25 mg/ml DNase I (Roche), for 40 min at 37°C. Released cells were collected, filtered through a 100 µM nylon mesh, and subjected to erythrolysis. Subsequently, cells were exposed to percoll gradient using 1.115 g/ml high-density and 1.06 g/ml of low-density percoll, and centrifuged at 1,350 g for 30 min at 4°C. Cells were collected from low-density interface, labeled with respective antibodies, and sp–DCs were sorted as CD11c^hi^Lin^−^ (Lin is defined as CD90, CD49b, F4/80, and CD19) on ARIA II or MoFlo.

### Cell Culture

Cells were cultured in RPMI 1640 medium supplemented with penicillin (50 U/ml), streptomycin (50 U/ml), sodium pyruvate (1 mM), β-mercaptoethanol (50 µM) (all purchased from Life Technologies), HEPES (25 mM) (Biochrom), and 10% fetal calf serum (Biochrom). Culture conditions were 37°C, 5% CO_2_, and 96-U bottom plates (Sarstedt).

### *In Vitro* Generation of Alloantigen-Induced Tregs

For the generation of allo-iTregs, 1 × 10^5^ CD4^+^Foxp3^RFP−^ peripheral T cells from Foxp3^RFP^ reporter mice (C57BL/6) were co-cultured with sp-DCs (BALB/c) in APC to T cell ratio of 1:10 in presence of 100 ng/ml IL-2 (R&D systems) for 6 days as described previously (31). TGF-β (5 ng/ml, R&D systems), vitamin C (0.1 mg/ml, Sigma), and RA (100 nM, Sigma) were added to the cultures as indicated. On day 6, allo-iTregs were either phenotypically analyzed or sorted on ARIA II or MoFlo using RFP as reporter. Sorted cells were used for further analyses.

### *In Vitro* Stability Assay

1 × 10^5^ sorted allo-iTregs were cultured for 2 days in untreated flat-bottom 96-well plates (Thermo Scientific), which were coated with anti-CD3 and anti-CD28 antibodies (1 µg/ml each, BioLegend). Afterward, cells were transferred into tissue-culture treated flat-bottom 96-well plates (Sarstedt) and cultured in presence of 50 ng/ml IL-2 (R&D Systems) until day 5. At the end of the culture, cells were analyzed for Foxp3 expression using RFP as reporter.

### Skin Transplantation and Adoptive Cell Transfer

To study the ability of allo-iTregs to prevent rejection of an allogeneic skin graft, naïve T cells (CD4^+^CD90.2^+^CD45.1^+^CD25^−^CD44^−^CD62L^hi^Foxp3^hCD2−^) were sorted from congenic CD45.1 Foxp3^hCD2^ reporter mice (C57BL/6), and 2.5 × 10^5^ sorted cells were injected intravenously into Rag2^−/−^ mice (C57BL/6 background) either with or without 5 × 10^5^ allo-iTregs (CD4^+^CD90.2^+^Foxp3^RFP+^) generated as described above. The following day, mice were anesthetized by intraperitoneal injection of Ketanest (10 mg/ml) and Rompun (0.2%) (10 µl/g bodyweight). Full thickness tail skin (1–1.5 cm^2^) from BALB/c mice (or from C57BL/6 mice in case of graft survival controls) was transplanted onto the lateral flank of Rag2^−/−^ recipient mice. The wound was applied with an ointment (Branolind^®^, Hartmann) and protected firstly with an elastic bandage (Rancolast^®^, Lohmann & Rauscher). Following this, a soft tape (Leukopor^®^) was put and finally the dressing was done using surgical tape (Blenderm™). Rapamycin was injected intraperitoneally for three times on days −1, 0, and 2 at 90 μg/mouse. The bandage was carefully removed after 9 days. Subsequently, the graft was monitored every 2 days for rejection characteristics. Mice were monitored for graft survival until day 100.

### DNA Methylation Analysis

Genomic DNA was isolated from sorted cells using the NucleoSpin XS kit (Macherey-Nagel) following the manufacturer’s protocol. The DNA concentration was quantified with a Nanodrop 1000 spectrophotometer (Peqlab). Methylation analysis of the TSDR and other Treg-specific epigenetic signature genes was performed using bisulfite sequencing as described previously (28). Only cells from male mice were used for the methylation analysis.

### RNA-Seq

Total RNA was isolated from sorted allo-iTregs using the RNeasy Plus Mini kit (Qiagen) according to the manufacturer’s protocol. Quality and integrity of total RNA was controlled on Agilent Technologies 2100 Bioanalyzer (Agilent Technologies). The RNA sequencing library was generated from 500 ng total RNA using Dynabeads^®^ mRNA DIRECT™ Micro Purification Kit (Thermo Fisher) for mRNA purification followed by ScriptSeq v2 RNA-Seq Library Preparation Kit (Epicenter) according to manufacture’s protocols. The libraries were sequenced on Illumina HiSeq2500 using TruSeq SBS Kit v3-HS (50 cycles, single ended run) with an average of 3 × 10^7^ reads per RNA sample. The sequenced libraries were assessed for read quality with *FastQC*.^1^ Quality assessment showed neither insufficient read quality nor nucleotide frequency biases introduced by primer contamination. Therefore, libraries were directly aligned versus mouse reference genome (assembly: GRCm38) using splice junction mapper *Tophat2* v2.1.1 (44) with default parameterization. Reads aligned to annotated genes were quantified with *htseq-count*^2^ program and determined read counts served as input to *DESeq2* (45) for detection and quantification of differential gene expression. In addition, RPKM (reads per kilobase max. transcript length per million mapped reads) values were computed for each library from raw gene counts and a principal component analysis (PCA) for all genes with variability based on variance among samples of scaled and mean centered rlog transformed count values was performed using base functions *scale* and *prcomp* from the statistical data analysis framework *R*. The list of *DESeq2* determined differentially expressed genes was filtered with an absolute log_2_ fold change (FC) cutoff of at least 1.0 and a *p*-value cutoff, corrected for multiple testing, of at most 0.05. RNA-seq data can be accessed under GEO/SRA accession number GSE96960.

### Statistics

For statistical comparison and *p*-value calculation of unmatched groups non-parametric Mann–Whitney test (two-tailed, confidence intervals = 95%) was performed. Survival curves were calculated using Kaplan–Meier analysis and the *p*-values were calculated with long-rank Mantel–Cox test. *p-*values <0.05 were considered significant (*p < 0.05, **p < 0.01, ***p < 0.001). The statistical analysis was performed using the Graph Pad Prism software.

## Results

### Increased Frequency of Allo-iTregs Showing Higher Foxp3 Expression Levels upon Addition of Vitamin C

**Figure 1:**
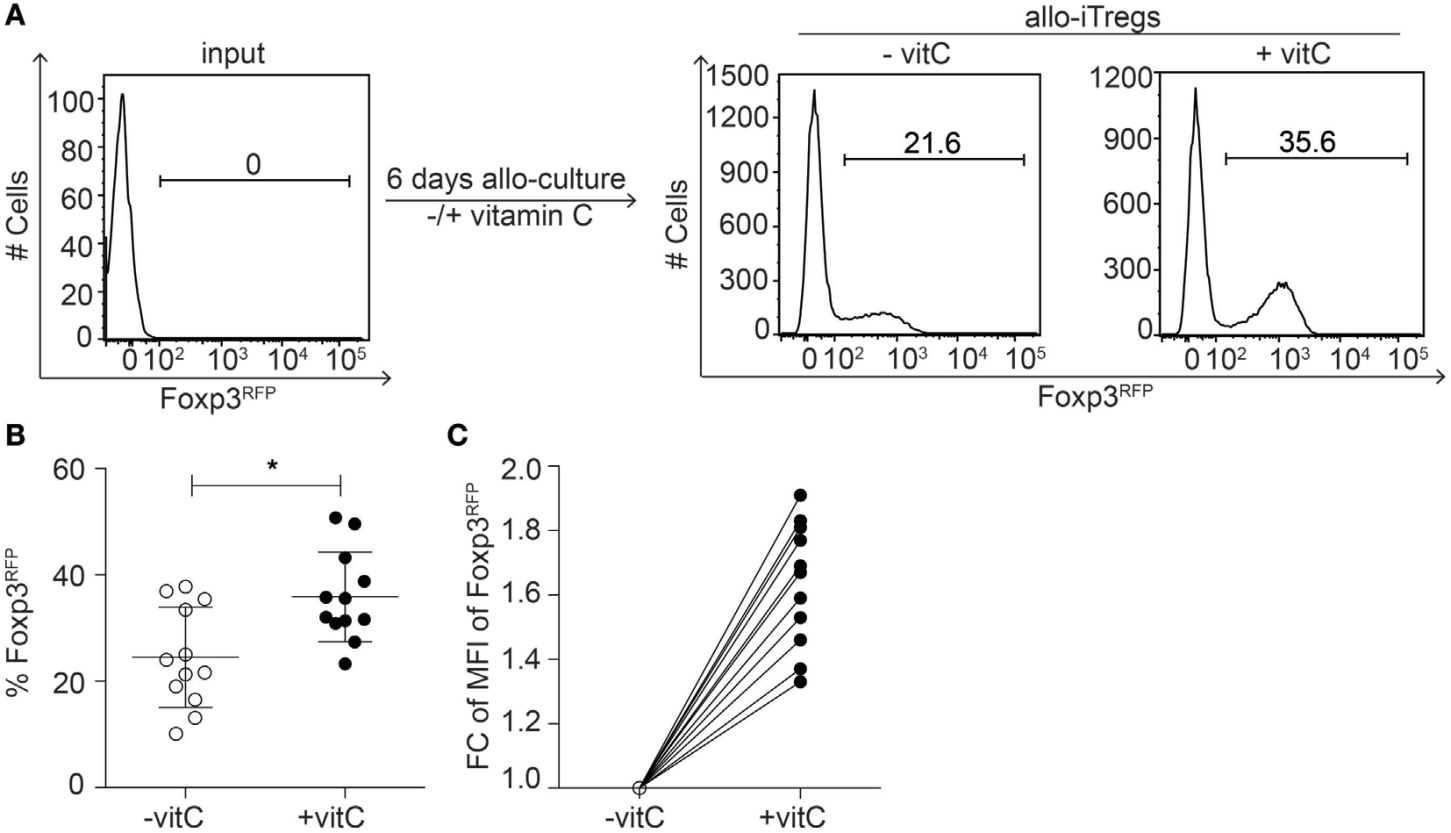
Addition of vitamin C results in increased frequency of allo-iTregs with higher Foxp3 expression levels. **(A)** Isolated sp-DCs from BALB/c mice were co-cultured with allogeneic CD4^+^Foxp3^RFP−^ peripheral T cells isolated from Foxp3^RFP^ reporter mice (C57BL/6) in presence of IL-2, TGF-β, and retinoic acid (RA) for 6 days. In part of the cultures, vitamin C was added. On day 6, expression of Foxp3 was analyzed by flow cytometry. Numbers indicate frequency of Foxp3^+^ cells. Representative data from one out of twelve independent experiments are depicted. **(B)** Graph shows frequency of Foxp3^+^ allo-iTregs from cultures ± vitamin C. Data are summarized from twelve independent experiments (mean ± SD) and tested for significance using Mann–Whitney test; *p < 0.05. **(C)** Graph shows fold change (FC) of mean fluorescent intensity (MFI) of Foxp3 expression between vitamin C-treated and non-treated allo-iTregs; lines connect data generated in the same experiment.

Previous studies have demonstrated that allo-iTregs are promising immunotherapeutic candidates to prevent allotransplant rejections (10); however, the long-term maintenance of the Treg lineage identity is a critical aspect. Since vitamin C had been recently shown to foster the generation of stable polyclonal Tregs (30, 42), we here aimed to study the impact of vitamin C on the *in vitro* generation of stable allo-iTregs. Thereto, peripheral CD4^+^Foxp3^−^ T cells were sorted from Foxp3^RFP^ reporter mice (C57BL/6) and cultured together with sorted CD11c^high^ sp-DCs from BALB/c mice (ratio 1:10 of sp-DCs:T cells) in the presence of exogenous IL-2, TGF-β, and RA. In part of the cultures, vitamin C was added from the beginning. When Foxp3 expression was analyzed in cultured T cells by flow cytometry at day 6, we observed that the addition of vitamin C led to a small, but significant increase in the frequency of Foxp3^+^ allo-iTregs when compared to cultures without vitamin C (Figures 1A,B). Furthermore, also slightly higher Foxp3 expression levels per cell were induced upon addition of vitamin C (Figure 1C), while the overall proliferation rate remained unaffected (data not shown). Together, these findings suggest that vitamin C fosters the *in vitro* generation of allo-iTregs leading to a higher frequency of Tregs that additionally express higher levels of Foxp3 protein.

### RNA-Seq Analysis Reveals a Similar Gene Expression Profile in Vitamin C-Treated and Non-Treated Allo-iTregs

**Figure 2:**
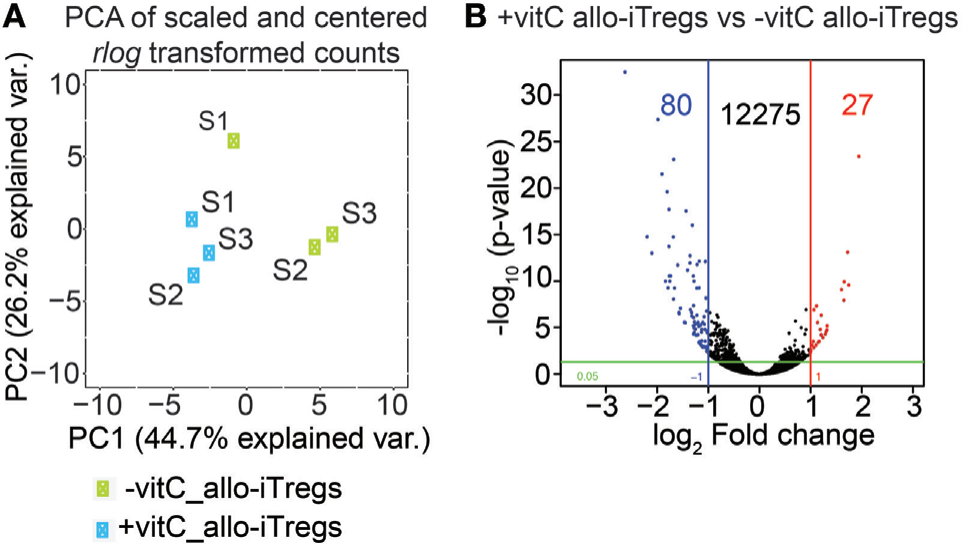
Vitamin C has only little influence on transcriptome of allo-iTregs. On day 6, Foxp3^RFP+^ cells were sorted from allo-iTreg cultures (±vitamin C), and total RNA was isolated from these cells for RNA-seq analysis. **(A)** Principal component analysis (PCA) of mean centered and scaled rlog-transformed read count values of RNA-seq data from −vitamin C allo-iTregs and +vitamin C allo-iTregs. Three replicates (S1, S2, and S3) are depicted for each condition (green symbols: −vitamin C allo-iTregs; blue symbols: +vitamin C allo-iTregs). **(B)** Volcano plot indicating number of differentially expressed genes between –vitamin C allo-iTregs and +vitamin C allo-iTregs according to the following criteria: |log_2_ (FC)| ≥ 1 and *p*-value ≤ 0.05. Data were generated from three independent experiments.

Having shown that vitamin C-treated allo-iTregs display slightly elevated Foxp3 expression levels, we next asked if the addition of vitamin C would also have an impact on their transcriptome. To this end, we generated allo-iTregs in the presence or absence of vitamin C as described above, sorted Foxp3^RFP+^ cells from the corresponding cultures and performed RNA-seq analyses. Global inspection of the data by principal component analysis revealed the existence of two closely related, but still separated clusters of sample replicates (Figure 2A). In total, 12,382 transcripts could be identified; however, only a very small number of these transcripts were differentially expressed (|log_2_ (FC)| ≥ 1 and *p*-value ≤0.05). 80 transcripts (e.g., *Frmd5, Dock4, Il10*, and *Plcd4*) were up- and 27 downregulated (e.g., *Cdh2, Gad2*, and *Adgra3*) when vitamin C-treated allo-iTregs were compared to allo-iTregs from cultures without vitamin C (Figure 2B; Table S1 in Supplementary Material). These data, which are nicely in line with recently published microarray data on polyclonal iTregs (42), suggest that vitamin C does not have a significant impact on the transcriptome of allo-iTregs. Strikingly, when we focused on previously identified Treg-specific signature genes (46), only seven signature genes (e.g., *Gpr15*) were up- and only two signature genes (*Hmgn3, Cd83*) downregulated upon addition of vitamin C (Table S2 in Supplementary Material). Confirming the flow cytometry data, mRNA levels of *Foxp3* were increased in vitamin C-treated allo-iTregs with a log_2_ FC of 0.8, similarly to *Il2ra* (CD25), whose mRNA levels were elevated in the vitamin C-treated group with a log_2_ FC of 0.6 (Table S2 in Supplementary Material). *Ctla-4, Gitr*, and *Eos*, the so-called Treg-specific epigenetic signature genes (22) as well as *Helios* were expressed but not differentially regulated between the two groups (Table S2 in Supplementary Material and data not shown). Together, these data indicate that vitamin C does not have a significant impact on the transcriptome of allo-iTregs.

### Augmented Demethylation at the TSDR and Other Treg-Epigenetic Signature Genes in Vitamin C-Induced Foxp3^+^ Allo-iTregs

**Figure 3:**
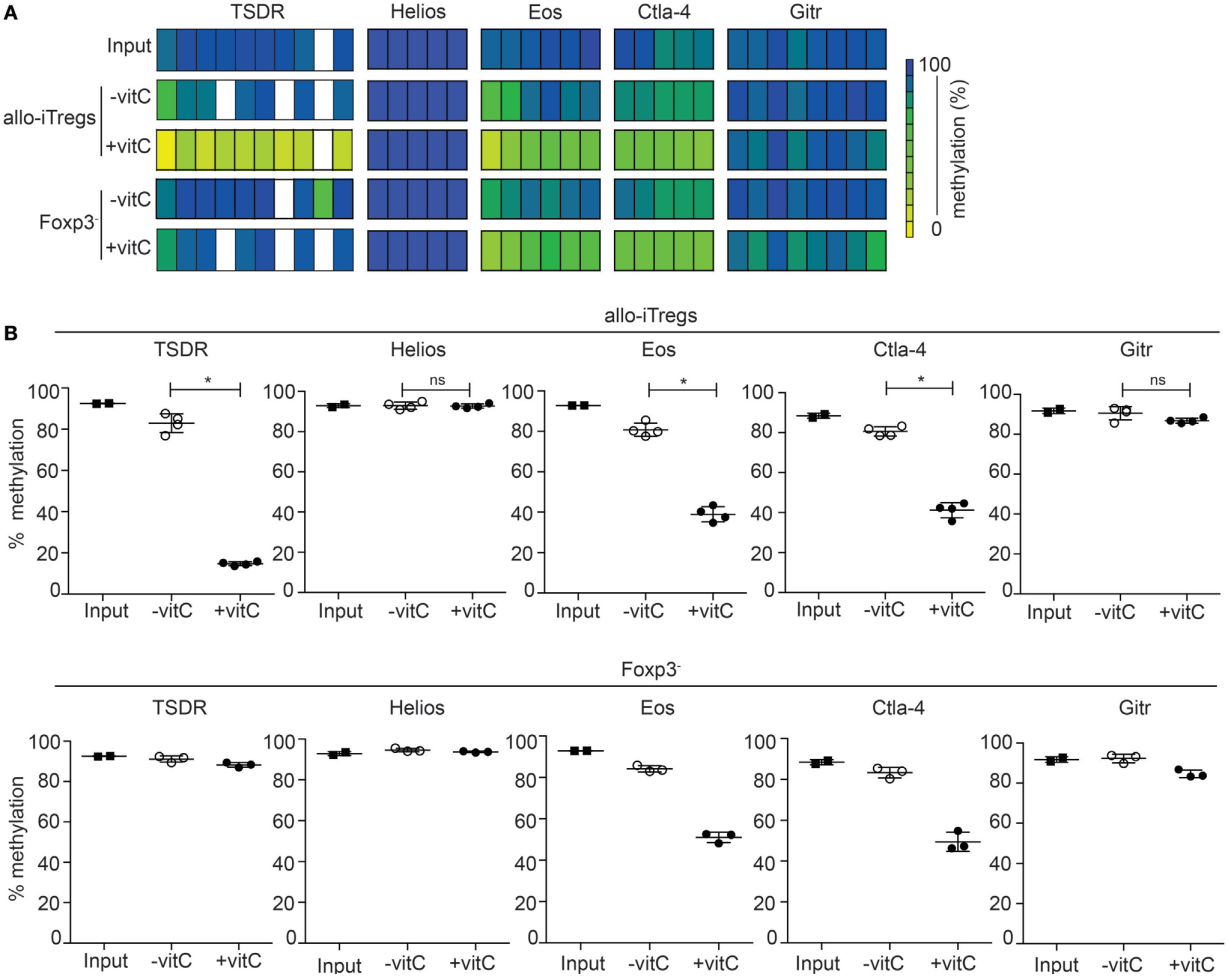
Addition of vitamin C augments demethylation at the TSDR and other Treg-epigenetic signature genes in allo-iTregs. On day 6, Foxp3^RFP+^ and Foxp3^RFP−^ cells were sorted from allo-iTreg cultures (±vitamin C), and genomic DNA was isolated from these cells for analysis of the methylation status of TSDR, *Helios, Eos, Ctla-4*, and *Gitr*. Genomic DNA from input cells (Foxp3^−^ peripheral CD4^+^ T cells) was analyzed as control. **(A)** Data from one out of four (allo-iTregs), three (Foxp3^−^ cells), and two (input cells) independent experiments are depicted. Each bar represents an individual CpG motif. Percentage of methylation is color-coded according to the scale. **(B)** Graphs show the average methylation status of all CpG motifs analyzed within indicated loci in sorted allo-iTregs (upper row) and corresponding Foxp3^−^ cells (lower row). Data are summarized from four (allo-iTregs), three (Foxp3^−^ cells), and two (input cells) independent experiments (mean ± SD). Data for allo-iTregs were tested for significance using Mann–Whitney test; *p < 0.05; ns, not significant.

The TSDR is selectively demethylated in *ex vivo*-isolated Tregs, but methylated in *in vitro* TGF-β induced Tregs (20, 22–24). Having shown that addition of vitamin C to the co-cultures leads to higher induction of Foxp3^+^ allo-iTregs, we next analyzed the effect of vitamin C on the methylation status of the TSDR and the other Treg-specific epigenetic signature genes. Thereto, Foxp3^+^ allo-iTregs and also corresponding Foxp3^−^ cells were sorted after 6 days of culture. Input cells (Foxp3^−^ peripheral CD4^+^ T cells) were used as control. Genomic DNA was isolated from these cell populations, bisulfite treated, and analyzed by pyrosequencing. As expected, the input cells were fully methylated at the TSDR and all other Treg-specific epigenetic signature genes (Figures 3A,B). In line with previous studies (30, 42), the addition of vitamin C led to a significant demethylation of the TSDR, *Eos*, and *Ctla–4* in Foxp3^+^ allo-iTregs, while in the absence of vitamin C hardly any demethylation was observed (Figures 3A,B). Interestingly, in presence of vitamin C also Foxp3^−^ cells from the alloantigen-specific Treg induction cultures showed a substantial demethylation of *Eos* and *Ctla–4*, whereas the TSDR methylation status remained largely unaffected (Figures 3A,B). In contrast to *Eos, Ctla-4*, and TSDR, *Gitr* and *Helios* were not substantially demethylated in any of the cell populations tested. Taken together, these data suggest that the effect of vitamin C on the TSDR methylation status is restricted to Foxp3^+^ cells and, apart from the TSDR, two additional Treg-specific epigenetic signature genes, namely *Eos* and *Ctla-4*, become demethylated upon addition of vitamin C, and therefore render vitamin C-treated allo-iTregs epigenetically more similar to “natural” Tregs.

### Vitamin C-Treated Allo-iTregs Maintain Foxp3 Expression *In Vitro*

**Figure 4:**
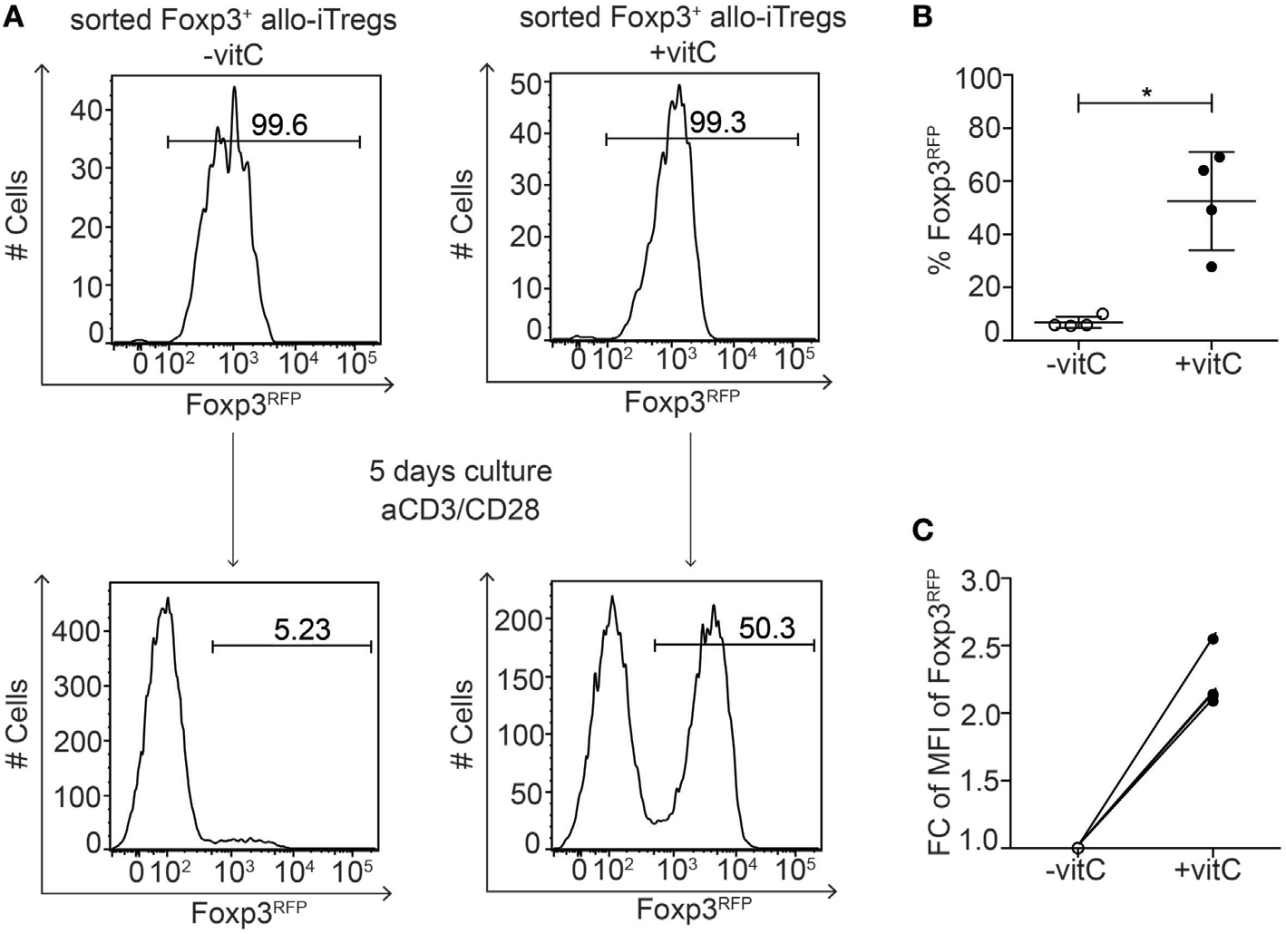
Vitamin C-treated allo-iTregs show increased Foxp3 stability after re-stimulation *in vitro*. **(A)** On day 6, Foxp3^RFP+^ cells were sorted from allo-iTreg cultures (±vitamin C), and re-stimulated *in vitro* with anti-CD3/anti-CD28 antibodies. On day 5 of the re-stimulation cultures, stability of Foxp3 expression was analyzed by flow cytometry. Representative data from one out of four independent experiments are depicted. **(B)** Graph shows frequency of Foxp3^RFP+^ cells after re-stimulation of allo-iTregs derived from cultures ±vitamin C. Data are summarized from four independent experiments (mean ± SD) and tested for significance using Mann–Whitney test; *p < 0.05. **(C)** Graph shows fold change (FC) of mean fluorescent intensity (MFI) of Foxp3 expression in Foxp3^RFP+^ cells after re-stimulation of allo-iTregs derived from cultures ±vitamin C; lines connect data generated in the same experiment.

Having shown that addition of vitamin C to the alloantigen-specific Treg induction cultures resulted in a pronounced demethylation of the TSDR in Foxp3^+^ allo-iTregs, we next asked whether vitamin C-treated allo-iTregs would display a stabilized Foxp3 expression since TSDR demethylation had been shown to be mandatory for the long-term maintenance of Foxp3 expression (15, 20, 22, 23, 25, 26). Hence, Foxp3^+^ allo-iTregs were induced in the presence or absence of vitamin C. At the end of the culture, Foxp3^+^ allo-iTregs were sorted to high purity and subsequently re-stimulated with plate-bound anti-CD3/anti-CD28 antibodies in the absence of exogenous TGF-β for 2 days, followed by culture for another 3 days on uncoated plates in the presence of IL-2 and succeeding assessment of Foxp3 stability by flow cytometry. In line with previous findings (20, 22, 30, 42), Foxp3^+^ allo-iTregs generated in the absence of vitamin C almost completely lost Foxp3 expression upon re-stimulation in the absence of exogenous TGF-β, while vitamin C-treated allo-iTregs showed a significantly increased stability of Foxp3 expression (Figures 4A,B). Moreover, again higher Foxp3 expression levels per cell were observed in vitamin C-treated allo-iTregs after re-stimulation (Figure 4C). These findings indicate that vitamin C supports the *in vitro* generation of allo-iTregs with stable Foxp3 expression.

### Vitamin C-Treated Allo-iTregs Prolong the Survival of an Allo-Skin Graft

**Figure 5:**
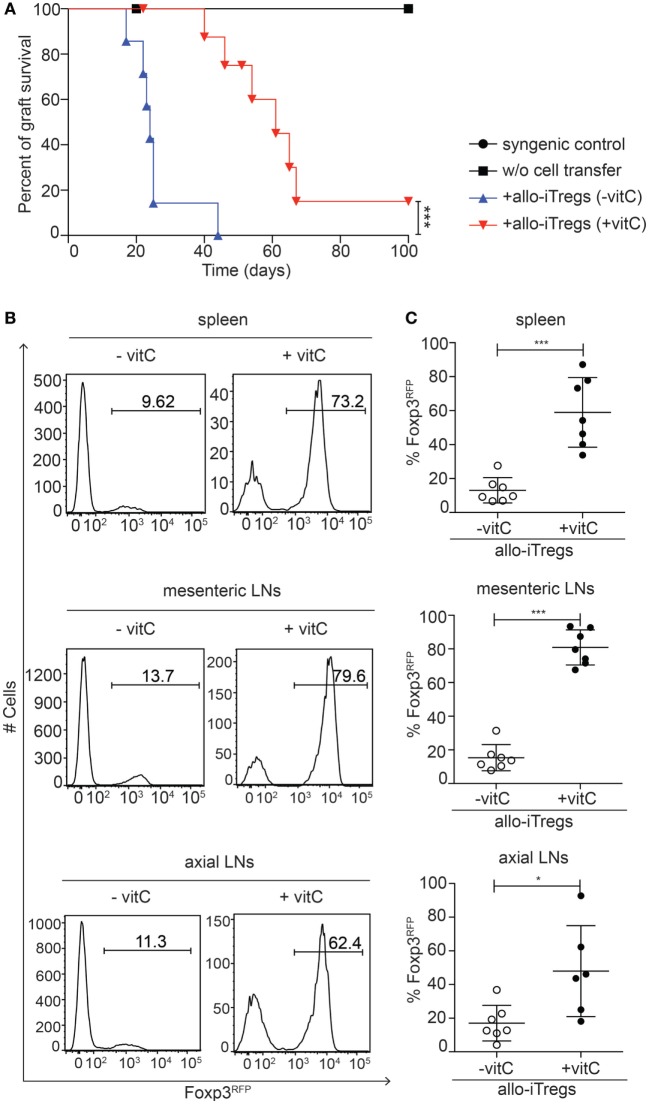
Vitamin C-treated allo-iTregs can efficiently prolong skin allograft survival. One day before skin transplantation, Foxp3^RFP+^ cells (CD45.2^+^) were sorted from allo-iTreg cultures (±vitamin C) and adoptively transferred together with freshly isolated CD4^+^ naïve T cells (CD45.1^+^) into Rag2^−/−^ (C57BL/6) mice. One day later, mice received an allogeneic skin transplant (BALB/c) and graft survival was monitored over a period of 100 days. Rag2^−/−^ mice receiving a syngeneic (C57BL/6) or allogeneic (BALB/c) skin transplant without adoptive T cell transfer were taken as graft survival controls. **(A)** Graph depicts percentage of graft survival for each group, and data are summarized from two independent experiments; syngeneic graft survival control (*n* = 2, black circle), allogeneic graft survival control (*n* = 3, black diamond), + allo-iTregs (−vitamin C) (*n* = 7, blue triangle) and + allo-iTregs (+vitamin C) (*n* = 9, red triangle). Data were tested for significance using the long-rank (Mantel cox) test; ***p < 0.001. **(B)** Upon graft rejection or on day 100, Foxp3 expression among adoptively transferred allo-iTregs within spleen, mesenteric, and axial lymph nodes (LN) was analyzed by flow cytometry. Representative histograms from indicated organs show frequency of Foxp3^RFP+^ cells among CD3^+^CD4^+^CD45.1^−^ cells. **(C)** Graphs show frequency of Foxp3^RFP+^ cells among CD3^+^CD4^+^CD45.1^−^ cells in indicated groups, and each dot represents an individual mouse. Data are summarized from two independent experiments (mean ± SD) and tested for significance using Mann–Whitney test; *p < 0.05; ***p < 0.001.

Encouraged by the finding that vitamin C-treated allo-iTregs showed an increased *in vitro* stability of Foxp3 expression, we next tested their *in vivo* suppressive capacity in a highly immunogenic allogeneic skin transplantation model. To this end, allo-iTregs (CD45.2^+^) were generated in presence or absence of vitamin C and adoptively transferred together with CD4^+^ naïve T cells (CD45.1^+^) into Rag2^−/−^ (C57BL/6) mice (day −1). At day 0, these mice received an allogeneic skin transplant (BALB/c) and graft survival was monitored over a period of 100 days. All mice were treated with rapamycin at the time of transplantation (days −1, 0, and 2). Rag2^−/−^ mice receiving only a syngeneic (C57BL/6) or allogeneic (BALB/c) skin transplant without adoptive T cell transfer were taken as graft survival controls, while Rag2^−/−^ mice adoptively transferred with CD4^+^ naïve T cells and receiving an allogeneic (BALB/c) skin transplant served as graft rejection controls. As expected, all graft survival controls accepted the grafts for the whole observation period (Figure 5A), while all graft rejection controls rapidly rejected the grafts (median survival time 45 days; data not shown). Mice adoptively transferred with allo-iTregs generated in absence of vitamin C showed an early graft rejection starting around day 20 (median survival time 24 days). Interestingly, vitamin C-treated allo-iTregs were able to significantly prolong the graft survival (median survival time 61 days), demonstrating that they display a higher suppressive ability *in vivo* (Figure 5A). At the end point for each individual transplanted mouse, the spleen, axial lymph nodes, and mesenteric lymph nodes were isolated and analyzed by flow cytometry for the *in vivo* stability of Foxp3 expression among adoptively transferred allo-iTregs. In all secondary lymphoid organs tested, vitamin C-treated allo-iTregs showed a significantly higher frequency of Foxp3^+^ cells when compared to allo-iTregs generated in absence of vitamin C, indicating an increased *in vivo* stability of vitamin C-treated allo-iTregs (Figures 5B,C) that overall results in a higher frequency of Foxp3^+^ Tregs among total CD4^+^ T cells (Figure S1 in Supplementary Material). Taken together, our data suggest that the addition of vitamin C to the alloantigen-specific Treg induction cultures leads to the generation of stabilized allo-iTregs, which display a higher *in vivo* suppressive ability and are able to significantly prolong the survival of highly immunogenic skin grafts.

## Discussion

In the recent past, the use of Tregs as an immunosuppressive therapy in transplantation medicine has gained a lot of interest, and several studies and clinical trials aimed to *in vitro* generate and expand sufficient numbers of stable Tregs, allowing long-term survival of the graft (47–51). Current studies focus on the use of alloantigen-specific Foxp3^+^ Tregs, and it has been demonstrated that these cells harbor a number of advantages compared to polyclonal Tregs (52–57). Most importantly, fewer cells are required for efficient suppression as their action is highly specific and locally restricted (53, 54). Thereby, the unwanted side effects of non-specific immunosuppression, like cancer and relapsing infections, can be significantly reduced. Another key feature regarding the applicability of Tregs in modern transplantation medicine is the long-term suppressive capacity of Tregs. To achieve this, stabilized Foxp3 expression within the Tregs is essential, and therefore improved protocols for the *in vitro* generation of sufficient numbers of allo-iTregs with stable Foxp3 expression need to be developed. Previously published studies had shown that TGF-β-induced polyclonal Tregs upon adoptive transfer *in vivo* lose Foxp3 expression and convert into effector T cells (21, 22), which under certain circumstances can even have detrimental effects. It is widely accepted that stability of Foxp3 expression is under epigenetic control and regulated by the methylation status of the TSDR (25). Recently, it was shown that TET enzymes play an important role for TSDR demethylation, and vitamin C acts synergistically with TET enzymes in this demethylation process (29, 30, 32–34), similarly to what has been reported for mouse embryonic stem cells (38) and precursors of erythrocytes (58). Interestingly, treatment of human embryonic stem cells with vitamin C led to the demethylation of almost 2,000 genes (59), and terminally differentiated B cells were able to convert into pluripotent stem cells (iPSCs) with signs of genome-wide demethylation (60). Although it has been recently demonstrated that both *in vitro* and *in vivo* generated allo-iTregs can acquire Treg-specific epigenetic patterns and prevent rejection of skin allografts (61), the impact of vitamin C on this process has not been reported so far. Thus, in the present study, we aimed to exploit the “demethylating activity” of vitamin C to *in vitro* generate allo-iTregs with stable Foxp3 expression, thereby ensuring their long-term suppressive ability even under inflammatory conditions which typically prevail in transplant recipients.

The presence of vitamin C in the alloantigen-specific Treg induction cultures not only led to an increased generation of Foxp3^+^ allo-iTregs, but most importantly also caused a pronounced TSDR demethylation, finally resulting in an elevated Foxp3 stability after re-stimulation *in vitro*. Importantly, Foxp3^−^ cells derived from the very same cultures did not show any signs of a pronounced TSDR demethylation, suggesting that vitamin C is not simply inducing the demethylation of all methylation-sensitive genes, but only acts on transcriptionally active ones. This finding is in line with a recently published study analyzing the *Il17a* locus in polyclonal iTregs, where any signs of demethylation upon treatment with vitamin C were found (42). However, the possibility that vitamin C also acts on the epigenome in a DNA demethylation-independent manner, as recently described for the induction of IL-17A expression in *in vitro* generated Th17 cells (62), also has to be considered.

Although Foxp3 expression is critically required for the suppressive capacity of Tregs (12–15), epigenetic regulation of *Foxp3* alone is not sufficient for proper development of the Treg lineage. The establishment of a CpG hypomethylation pattern at other loci apart from *Foxp3* is required for Treg development, a process that is established in a Foxp3-independent manner. These epigenetically regulated genes in Tregs are called Treg-specific epigenetic signature genes and include *Eos, Ctla-4, Gitr*, and *Helios* (22, 27, 28, 63). Interestingly, the methylation analysis of these loci in Foxp3^+^ Tregs derived from alloantigen-specific Treg induction cultures revealed that the addition of vitamin C resulted in a significant demethylation of only *Eos* and *Ctla-4*, but not *Gitr* and *Helios*, suggesting that *Eos* and *Ctla-4* can be more easily epigenetically modified and that different conditions, e.g., a longer treatment with vitamin C, are required for the epigenetic imprinting of *Gitr* and *Helios*. The finding that *Eos* and *Ctla-4* became substantially demethylated even in Foxp3^−^ cells from the alloantigen-specific Treg induction cultures further supported this hypothesis.

Gene expression profiling of vitamin C-treated allo-iTregs compared to allo-iTregs generated in the absence of vitamin C revealed only minor differences between the two populations, indicating that under these conditions vitamin C has a significant impact mainly on the epigenome, but marginally on the transcriptome of these cells. Based on these results, we hypothesize that the differences between these two populations regarding their functional properties and Foxp3 stability are largely dependent on the vitamin C-induced epigenetic modifications. Our findings on allo-iTregs are in line with previously published microarray data of polyclonal Foxp3^+^ iTregs, where only 50 genes were found to be differentially regulated upon addition of vitamin C to the Treg induction cultures (42). However, other cell types, e.g., stromal cells, primary human dermal fibroblasts, or murine iPSCs, display a higher sensitivity toward vitamin C as revealed by pronounced alterations in gene expression upon cell culture supplementation with vitamin C (64–66). These findings indicate that in principle vitamin C is capable of inducing significant changes at the transcriptional level, thereby modulating development and differentiation of diverse cell types.

The addition of vitamin C to the Treg induction cultures not only resulted in a pronounced demethylation of the TSDR, *Eos*, and *Ctla-4*, but also significantly affected the stability of Foxp3 expression both *in vitro* and *in vivo*. Previous studies had already analyzed the stability of TGF-β induced iTregs and observed that these iTregs display an unstable Treg phenotype and rapidly lose Foxp3 expression and suppressive activity upon *in vitro* re-stimulation in the absence of TGF-β or upon adoptive transfer (20–22). Notably, the stability of Foxp3 expression could be increased upon *in vivo* administration of IL-2/anti-IL-2 complexes, resulting in enhanced TSDR demethylation (67). Furthermore, a recent study analyzing allo-iTregs generated upon co-culture of naïve CD4^+^ T cells from C57BL/6 mice with allogeneic APCs from BALB/c mice suggested that the addition of RA to the Treg induction cultures positively influences the stability of Foxp3 expression, although a direct side-by-side comparison of iTregs generated in the presence or absence of RA had not been performed (68). In the present study, the addition of RA was not sufficient to stabilize Foxp3 expression in allo-iTregs, suggesting that in cultures with highly pure CD11c^+^ sp-DCs as APCs the addition of both RA and vitamin C is a prerequisite to epigenetically remodel the *Foxp3* locus and stabilize Foxp3 expression. Importantly, the stabilization of Foxp3 expression directly translates into a higher *in vivo* suppressive capacity, as allo-iTregs generated in presence of vitamin C significantly prolonged the survival of an allo-skin graft, in contrast to allo-iTregs generated in absence of vitamin C, which failed to protect the graft. It is worth mentioning that even allo-iTregs generated in the presence of vitamin C could not achieve a long-term acceptance of the graft, most probably due to the highly immunogenic environment of the skin transplant and the full mismatch of major histocompatibility complexes between C57BL/6 and BALB/c mice. Nevertheless, vitamin C-treated allo-iTregs displayed a significantly increased stability of Foxp3 expression upon *in vivo* transfer, in line with two recently published studies analyzing the stability of Foxp3 expression in polyclonal iTregs generated in presence of vitamin C after adoptive transfer into Rag2^−/−^ or immunocompetent recipient mice (30, 42).

In conclusion, we could demonstrate that the addition of vitamin C to alloantigen-specific Treg induction cultures is a promising strategy for the generation of allo-iTregs, which are epigenetically more similar to “natural” Tregs, show a stabilized Foxp3 expression *in vitro* and *in vivo* under inflammatory conditions and thus are suitable candidates for future therapeutic applications.

## Data and Materials Availability

The RNA-seq data of the present study can be found via the following access link: https://www.ncbi.nlm.nih.gov/geo/query/acc.cgi?acc=GSE96960.

## Ethics Statement

The animal experiments were approved by the Niedersächsisches Landesamt für Verbraucherschutz und Lebensmittelsicherheit (LAVES): animal licensing committee permission no. 10/0071 and 15/1878. All experiments were performed in accordance with regulations according to FELASA, and animals were handled with care and welfare.

## Author Contributions

EN, MH-W, MH, and SF performed the experiments and interpreted the data. RG generated RNA-seq data. MB performed the RNA-seq data analysis. EJ interpreted the data from skin transplantation experiments and contributed to the manuscript. EN and JH designed the research, interpreted the data, and wrote the paper.

## Conflict of Interest Statement

The authors declare that the research was conducted in the absence of any commercial or financial relationships that could be construed as a potential conflict of interest.

## Footnotes

^1^http://www.bioinformatics.babraham.ac.uk/projects/fastqc.

^2^http://www-huber.embl.de/users/anders/HTSeq.

## Supplementary Material

Figure S1 One day before skin transplantation, Foxp3^RFP+^ cells (CD45.2^+^) were sorted from allo-iTreg cultures (±vitamin C) and adoptively transferred together with freshly isolated CD4^+^ naïve T cells (CD45.1^+^) into Rag2^−/−^ (C57BL/6) mice. One day later, mice received an allogeneic skin transplant (BALB/c) and graft survival was monitored over a period of 100 days. Upon graft rejection or on day 100, Foxp3 expression among total adoptively transferred CD4^+^ T cells within spleen, mesenteric, and axial lymph nodes (LN) was analyzed by flow cytometry. Graphs show frequency of Foxp3^+^ cells (CD45.2^+^Foxp3^RFP+^ plus CD45.1^+^Foxp3^hCD2+^) among total CD3^+^CD4^+^ cells in indicated groups, and each dot represents an individual mouse. Data are summarized from two independent experiments (mean ± SD) and tested for significance using Mann–Whitney test; ***p* < 0.01; ns, not significant.

Table S1 Sorted Foxp3^RFP+^ cells from allo-iTreg cultures (±vitamin C) were analyzed by RNA-seq. List of differentially expressed genes between −vitamin C allo-iTregs and +vitamin C allo-iTregs according to |log_2_ (FC)| ≥ 1 and *p*-value ≤0.05 is shown. The log_2_ fold change between the two groups, the RPKM counts of each replicate and *p*-values for all genes are depicted.

Table S2 Sorted Foxp3^RFP+^ cells from allo-iTreg cultures (±vitamin C) were analyzed by RNA-seq. List of genes from the Treg expression signature published by Hill et al. (46) is shown. The Affymetrix microarray probe set-based signature was translated into a gene-linked signature based on gene symbol. The procedure includes elimination of probe sets having no counterpart and links multiple gene-specific probes to the corresponding gene locus. Log_2_ fold change between −vitamin C allo-iTregs and +vitamin C allo-iTregs, DESeq-normalized counts of each replicate and *p*-values are depicted. Signature genes being significantly up- or downregulated upon addition of vitamin C are highlighted in bold.
