# Supplementary figures and images for: Alloantigen-Induced Regulatory T Cells Generated in Presence of Vitamin C Display Enhanced Stability of Foxp3 Expression and Promote Skin Allograft Acceptance
Source: Front Immunol. 2017 Jun 28;8:748. doi: 10.3389/fimmu.2017.00748 (PMC5487376; doi:10.3389/fimmu.2017.00748)

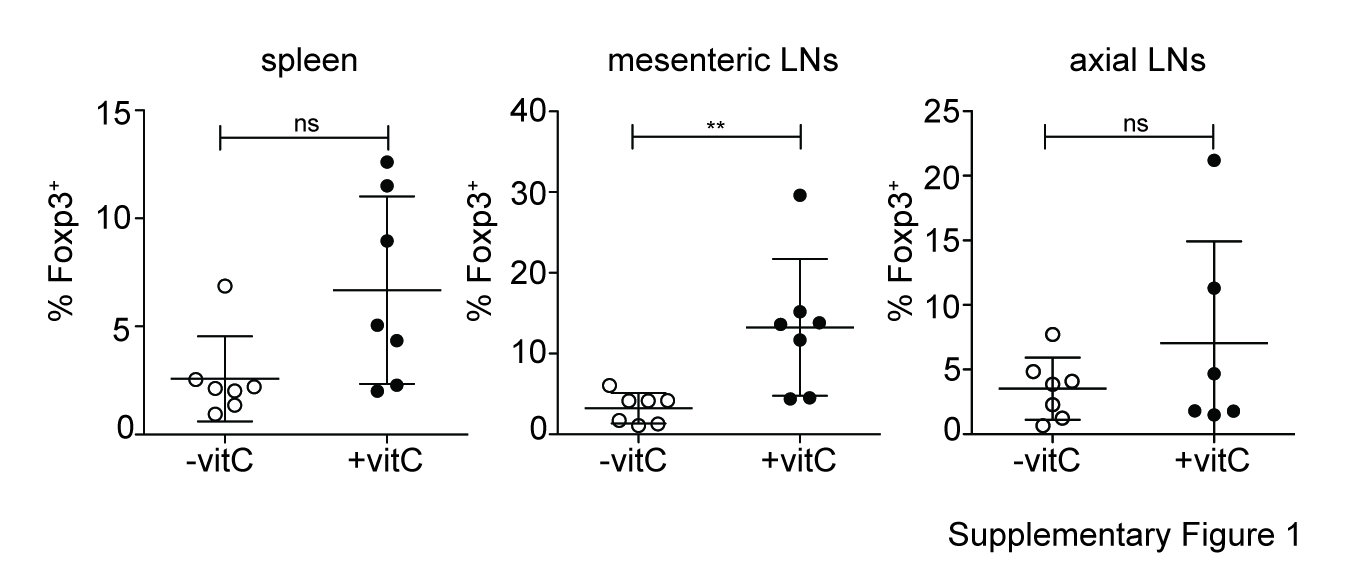

Supplement: Figure S1 — One day before skin transplantation, Foxp3RFP+ cells (CD45.2+) were sorted from allo-iTreg cultures (±vitamin C) and adoptively transferred together with freshly isolated CD4+ naïve T cells (CD45.1+) into Rag2−/− (C57BL/6) mice. One day later, mice received an allogeneic skin transplant (BALB/c) and graft survival was monitored over a period of 100 days. Upon graft rejection or on day 100, Foxp3 expression among total adoptively transferred CD4+ T cells within spleen, mesenteric, and axial lymph nodes (LN) was analyzed by flow cytometry. Graphs show frequency of Foxp3+ cells (CD45.2+Foxp3RFP+ plus CD45.1+Foxp3hCD2+) among total CD3+CD4+ cells in indicated groups, and each dot represents an individual mouse. Data are summarized from two independent experiments (mean ± SD) and tested for significance using Mann–Whitney test; **p < 0.01; ns, not significant. [file image_1.tif]
